# Camera realignment imposes a cost on laparoscopic performance

**DOI:** 10.1038/s41598-021-96965-6

**Published:** 2021-09-03

**Authors:** Christopher L. Hewitson, Sinan T. Shukur, John Cartmill, Matthew J. Crossley, David M. Kaplan

**Affiliations:** 1 School of Psychological Sciences, Perception in Action Research Centre, Centre for Elite Performance, Expertise and Training, Faculty of Medicine, Health and Human Sciences, Macquarie University, Australian Hearing Hub, Level 3, 16 University Drive, Sydney, NSW 2109 Australia; 2 Department of Surgery, College of Medicine, University of Kufa, Kufa, Iraq; 3 Department of Clinical Medicine, Faculty of Medicine, Health and Human Sciences, Macquarie University, Sydney, NSW 2109 Australia

**Keywords:** Motor control, Sensorimotor processing, Human behaviour

## Abstract

There is an unresolved question about whether realigned visual feedback is beneficial or costly to laparoscopic task performance. We provide evidence that camera realignment imposes a reliable cost on performance across both naive controls and experienced surgeons. This finding clarifies an important ongoing discussion in the literature about the effects of camera realignment, which could inform the strategies that laparoscopic surgeons use in the operating room.

## Introduction

A fundamental challenge routinely faced by laparoscopic surgeons is that, since an assistant can only direct the camera through a non-aligned port, the viewpoints of the surgeon and the laparoscopic camera are often considerably misaligned. This causes instrument movements in the workspace to produce counter-intuitive movements on the video display. One obvious coping strategy in such scenarios is to reposition the camera to precisely counteract the misalignment (henceforth called realignment). However, while this successfully realigns camera and surgeon viewpoints, it also introduces discrepancies between how instrument movements are visually depicted on the screen and the surgeon’s actual movements in the workspace. There is a tension in the literature with respect to the impact of camera realignment, with some research reporting that it is helpful^1–5^, and other research indicating that it is harmful^6–10^. A similar tension exists when viewing this issue from the perspective of motor learning neuroscience. Here, the dominant empirical observation is that visuomotor misalignments (or perturbations) are generally harmful to performance^11–14^. Yet it is currently unclear to what extent these findings—which relate to simple unimanual reaching and pointing—should be expected to apply to a laparoscopic context.

In the current study, we investigated the effects of realigned visual feedback on motor performance in a laparoscopic task for both naive controls and experienced surgeons. We observed that realignment imposed a reliable cost on task performance across both groups.

## Methods

10 expert surgeons and 10 naive controls participated in the study. All were right-hand dominant (LQ > 70) assessed using the ten-item version of the Edinburgh Handedness Inventory^15^, with normal or corrected to normal vision and no reported motor impairments. All surgeons (age 47 ± 14 years; 9 males, 1 female) were from Macquarie University Hospital, and had completed > 100 laparoscopic procedures according to self-report. Of those, 3 reported completing > 1000 MIS procedures. Controls (23 ± 3 years; 4 males, 6 females) were Macquarie University undergraduates with no prior surgical experience or training. The study was performed in compliance with the Declaration of Helsinki and was approved by the Macquarie University Human Research Ethics Committee (#5201800444). All participants provided informed written consent before taking part in the study.

**Figure 1:**
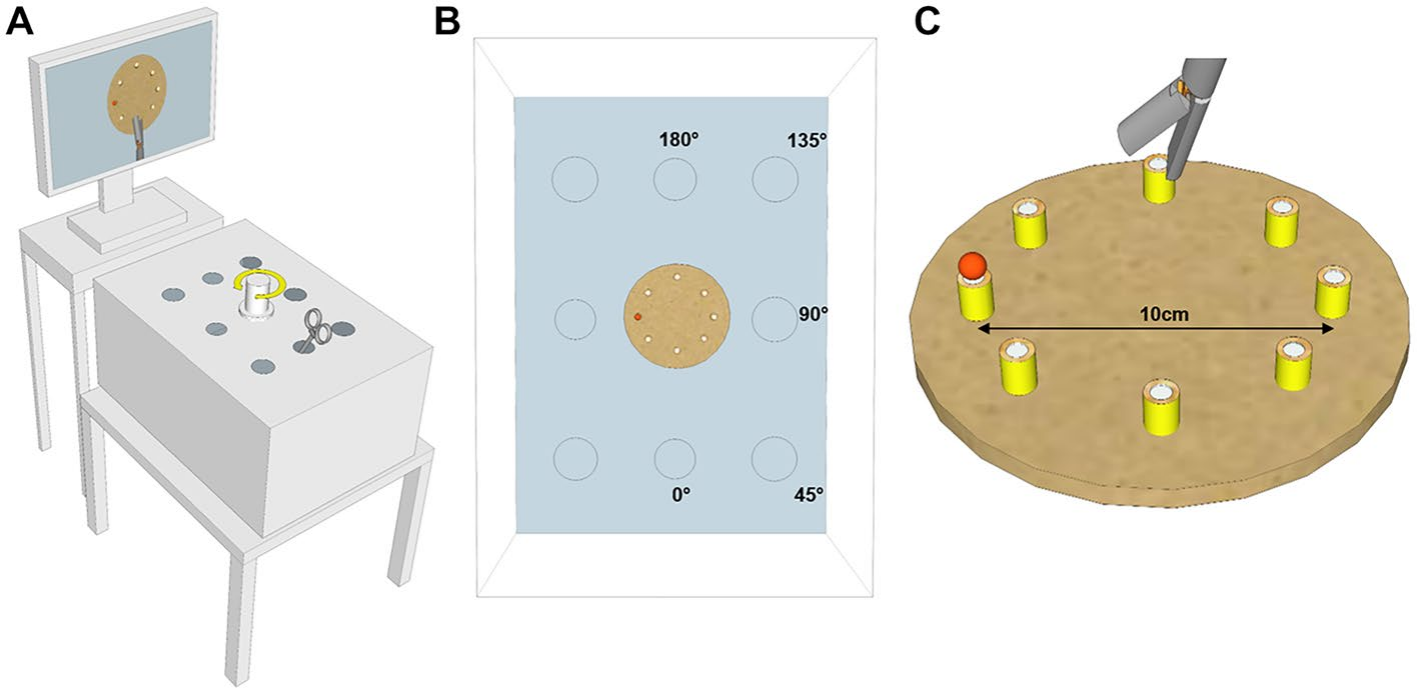
(**A**) Schematic of box trainer with custom adjustable camera mount used in the experiment. (**B**) Overhead view of the box trainer ports that were tested. Transparent walls are depicted for visualization purposes only and permit comparison of the port locations relative to the peg transfer task apparatus on the inner floor of the box trainer. (**C**) Close-up view of the peg transfer task apparatus.

Participants completed a unimanual version of the peg transfer task^16–20^ using a laparoscopic dissector (Ethicon Inc.) in a custom-built box trainer (Fig. 1A). Instrument ports were positioned at 45° increments around a centrally positioned camera that provided an overhead view of the workspace (Fig. 1B), similar to a number of previous studies of laparoscopic performance under camera rotation^9,10,21^. The camera was attached to a custom adjustable mount with interlocking teeth on both the camera and base that permitted 360° rotation in precise, highly reproducible 15° increments. The peg transfer apparatus consisted of 8 cylindrical pegs (1 cm × 0.5 cm) positioned equidistant from the center (5 cm radius) at 45° increments, and secured to a circular base (Fig. 1C). This configuration, which is invariant over angular rotations, was chosen to ensure that no overt visual cues about the experimental condition (i.e., camera rotation) were available to the participants during the experiment. A shallow high contrast white recess on the top of each peg provided a secure, easily visible target site for placement of a small orange foam ball (0.5 cm).

During the task, participants were instructed to transfer the ball as quickly and directly as possible back and forth between two opposing pegs. Visual feedback was displayed on a monitor mounted vertically and positioned directly in front of the participant. Instrument position was tracked using an infrared 3D motion tracking system (Polaris Vicra, Northern Digital Inc.) mounted above the box trainer, which recorded the positions of the infrared sensors of an NDI rigid body secured to the top of the tool handle at 20 Hz. Instrument tip position was extrapolated via the “pivot” function in the NDI ToolBox tracking software. Position data was exported as a csv file prior to analysis.

The overall experiment involved 2 groups (expert, control) × 2 conditions (canonical, realigned) × 9 ports (0°, 45°, 90°, 135°, 180°) × 10 trials as factors. Every factor except group was within-subject. In 5 *canonical* visual feedback conditions, transfers were completed from 5 different port locations (0°, 45°, 90°, 135°, 180°) with the camera rotated 0°. In 4 *realigned* visual feedback conditions (45°, 90°, 135°, 180°), the camera was rotated by an amount equal and opposite to the active port location. Different pegs were used for each condition such that start and end targets were always either at the rightmost (90°) or leftmost (270°) positions in visual space. This resulted in visual feedback about instrument and target position matched to the 0° (canonical) port irrespective of the instrument’s real position and orientation in the workspace, which was the same in every condition. Trials were blocked by port location, and port order was randomised and counterbalanced across participants. The canonical condition was always completed first at each port location. Kinematic measures including movement time(s), velocity (mm/s) and movement smoothness estimated by dimensionless total squared jerk (tsj)^22^ (a.u.) were calculated for all reaches. We performed mixed-design ANOVAs treating either movement time or movement smoothness as a dependent variable, group as a between-subject factor, and condition as a within-subject factor. We performed post-hoc t-tests to ascertain the direction of significant omnibus results. All analyses were performed using Pingouin 0.3.11^18^.

## Results

Camera realignment increased movement time for both groups to similar degrees as evidenced by a significant main effect of condition [*F*(1,18) = 32*.*95,* p* < 0*.*001,* η*^2^ = 0*.*65], and a non-significant group × condition interaction [*F*(1,18) = 0*.*57, *p* = 0*.*46,* η*^2^ = 0*.*03]. Post hoc t-tests support the direction of this effect [*t*(19*.*0) =  *− *5*.*81,* p* < 0*.*01,* d* =  *− *0*.*77]. Overall, experts moved more quickly than controls as evidenced by a main effect of group [*F*(1,18) = 9*.*62,* p* = 0*.*01,* η*^2^ = 0*.*35], and further supported by a post hoc t-test [*t*(18*.*0) = 3*.*1,* p* = 0*.*01,* d* = 1*.*39].

**Figure 2:**
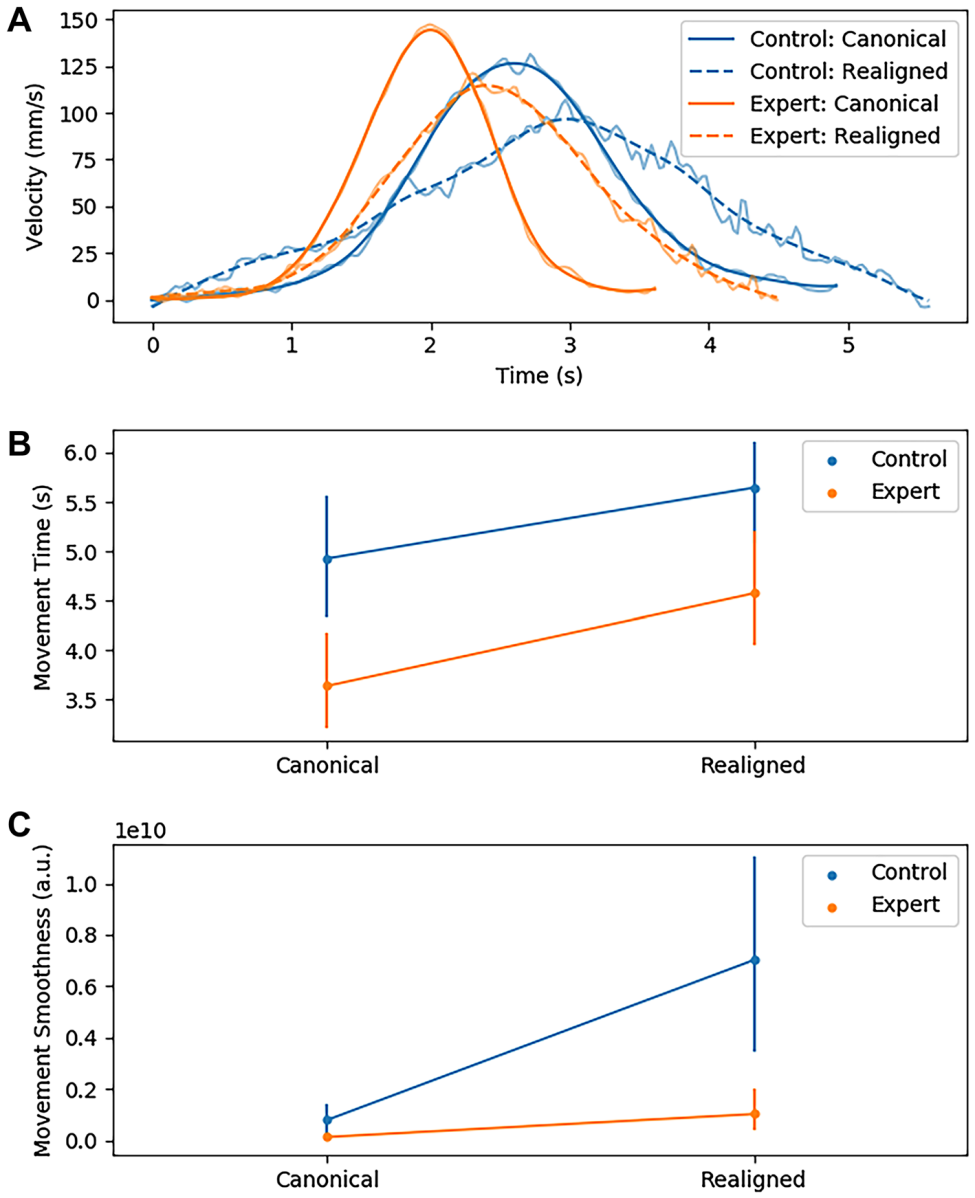
(**A**) Mean movement speed profiles for each group and condition. Semi-transparent lines are the raw signal and opaque lines are a smoothed version using a second order lowpass Butterworth filter with critical frequency 0.05. (**B**) Mean movement time for each group in each condition averaged across all ports. Error bars indicate 95% confidence intervals. (**C**) Mean movement smoothness (tsj) for each group in each condition averaged across all ports. Lower tsj indicates smoother movements. Error bars indicate 95% confidence intervals. The range of the y-axis is from 0 to 1*e*^10^.

Experts moved more smoothly than controls overall as evidenced by a significant main effect of group [*F*(1,18) = 9*.*09,* p* = 0*.*01,* η*^2^ = 0*.*34], and supported by a post hoc t-test [*t*(18*.*0) = 3*.*02,* p* < 0*.*01,* d* = 1*.*35]. Realignment decreased movement smoothness for both groups as evidenced by a significant main effect of condition [*F*(1,18) = 12*.*12,* p* < 0*.*001,* η*^2^ = 0*.*4], and supported by a post hoc t-test [*t*(19*.*0) =  *− *3*.*05,* p* < 0*.*01,* d* =  *− *0*.*9]. However, movement smoothness was more severely degraded for controls than for experts as evidenced by a significant group × condition interaction [*F*(1,18) = 6*.*79,* p* < 0*.*05,* η*^2^ = 0*.*27], and supported by a post hoc t-test comparing groups under realignment [*rot* × *group*: *t*(18*.*0) = 2*.*84,* p* < 0*.*05,* d* = 1*.*27]. See Fig. 2 for a visualization of these effects.

## Discussion

In this study, we showed that camera realignment leads to greater movement times to similar degrees for both experts and controls, and decreased movement smoothness for both groups, but significantly more for controls than for experts. Why might some previous studies indicate that camera realignment is helpful^1–5^, while others suggest that it is harmful^6–10^?

One possible explanation emerges from another challenge routinely faced by laparoscopic surgeons. Namely, since laparoscopic instruments are inserted through ports in the skin that serve as pivot points, instrument tip motion is reversed relative to hand motion—a complication referred to as the “fulcrum effect”^1^. Camera realignment may help as it causes instrument tip movements to *appear* to match hand movements. This suggests that it is primarily those that are not well-practised in dealing with the fulcrum effect that may benefit from realignment. In line with this, laparoscopic surgeons already acclimated to the fulcrum effect, do not appear to show similar improvements from camera realignment as naive controls^23^. (But see Johnston et al.^2^ for possible exceptions).

The broader literature on tool use reinforces this interpretation. In tool-based contexts similar to MIS, which transform hand movements into inverted movements of the tool tip, performance is improved when visual feedback is manipulated so there is a spatial correspondence between the location of the stimulus and the direction of tool tip motion, independent of the movement direction of the hand^6,24,25^. Importantly, the participants in these studies were all naive.

Misalignment between the viewpoint of the surgeon and the camera is an inherent challenge in laparoscopic surgery. Although simple camera realignment may be helpful under certain conditions and in some populations, the overall literature in combination with our current results, show that it is not a reliable intervention to improve performance—a finding that could usefully inform the strategies that laparoscopic surgeons use in the operating room. Instead, experienced laparoscopic surgeons seem capable of learning to cope with challenging misalignment without relying on camera counter-rotation. One possibility suggested by the broader literature on motor learning is that humans are capable of using cognitive strategies to respond rapidly and flexibly to changes in visuomotor mappings^26^. It is therefore possible that expert surgeons have discovered appropriate strategies for contending with camera misalignments and consequently do not require physical realignment of the camera to maintain stable performance. We suggest that a productive and still largely unexplored research direction is to understand what confers these surgeons with such remarkable adaptability including whether they successfully exploit high-level cognitive strategies or heuristics^27,28^.
